# POST introduction evaluation (PIE) of the malaria vaccine introduced in three pilot countries (Ghana, Kenya, and Malawi) in 2021

**DOI:** 10.1186/s12936-025-05590-5

**Published:** 2025-10-14

**Authors:** Jenny A. Walldorf, Gillian F. Mayers, Kwame Amponsa-Achiano, Rose Jalang’o, Mike Chisema, Lydia Khalayi, Josephine Njoroge, Adam Haji, Peter O. Tweneboah, Emmanuel T. Sally, Donnie Mategula, Brenda Mhone, Boston Zimba, Esther Chirwa, Naziru Tanko Mohammed, Michael Rockson Adjei, Jackson Sillah, Eliane Pellaux-Furrer, Mgaywa G. M. D. Magafu, Rafiq N. A. Okine

**Affiliations:** 1 Department of Immunization, Vaccines and Biologicals, World Health Organization, Geneva, Switzerland; 2 Independent Consultant, Echenevex, France; 3 Expanded Programme On Immunization, Public Health Division, Ghana Health Service, Accra, Ghana; 4 National Vaccines and Immunization Program, Nairobi, Kenya; 5 Ministry of Health, Lilongwe, Malawi; 6 World Health Organization, Nairobi, Kenya; 7 World Health Organization, Accra, Ghana; 8 Malawi-Liverpool Wellcome Programme, Blantyre, Malawi; 9 Kamuzu University of Health Sciences, Blantyre, Malawi; 10 Liverpool School of Tropical Medicine, Liverpool, UK; 11 World Health Organization, Lilongwe, Malawi; 12 Tropical and Vector-Borne Diseases Unit, World Health Organization Africa Regional Office, Brazzaville, Congo; 13 Vaccine-Preventable Diseases Unit, World Health Organization Africa Regional Office, Brazzaville, Congo; 14 Global Malaria Programme, World Health Organization, Geneva, Switzerland

**Keywords:** Malaria vaccine, Post-introduction evaluation, Malaria vaccine implementation programme, New vaccine introduction, Malaria vaccine pilot introduction, Ghana, Kenya, Malawi]

## Abstract

**Background:**

The World Health Organization (WHO) recommends the use of malaria vaccines for the prevention of *Plasmodium falciparum* malaria in moderate to high transmission areas, administered in a 4-dose schedule in children from 5 months of age. The vaccine is a ground-breaking new tool to add to the existing package of recommended malaria interventions to reduce malaria morbidity and mortality. Ghana, Kenya, and Malawi were the first countries to introduce the RTS,S/AS01_E_ (RTS,S) malaria vaccine into their childhood immunization programmes in 2019 as part of a pilot programme called the Malaria Vaccine Implementation Programme (MVIP).

**Methods:**

The WHO’s post-introduction evaluation (PIE) methodology was adapted to evaluate malaria vaccine implementation in each of the three pilot countries at least a year after the vaccine’s introduction. Semi-structured questionnaires were used to interview immunization staff at national, sub-national, and health facility levels, supplemented with systematic observations of vaccination sessions and vaccine storage sites. At the health facility, a sample of caregivers of eligible children was also interviewed. Sites were purposively selected to include a range of past immunization coverage and varied demographics among the populations served.

**Results:**

All three countries successfully introduced the malaria vaccine during the MVIP. Reported malaria vaccine median coverage at least 2 years after the start of the pilot ranged from 69–91% for dose 1, 62–82% for dose 2, to 58–81% for dose 3 by 24–30 months from the start of the pilot. Coverage for dose 4 was lower as fewer children were eligible during the PIE reporting timeframe. Best practices identified during the PIEs included: early involvement of subnational stakeholders; advance updating and distribution of recording and reporting tools to include malaria vaccine; pre-assessment of cold chain capacity and scale-up; investment of time and resources in health worker trainings and refreshers; involvement of community health workers; robust defaulter tracing mechanisms; ensuring community “dialogue” with continuity of advocacy, communication, and social mobilization activities after initial introduction; regular onsite supervisory visits before, during and after introduction; and use of social media for messaging.

**Conclusions:**

Malaria vaccine is an important intervention as part of a comprehensive malaria control strategy. Conducting a PIE is useful to identify best practices and lessons learned. New vaccination contacts take time to establish and achieve high coverage as communities become aware of and understand when, why, and how to access the malaria vaccine. The malaria vaccine was successfully introduced as part of the routine childhood immunization programme with strong intersectoral collaboration and planning, involving both immunization and malaria stakeholders, comprehensive training, and social mobilization efforts pre- and post-introduction.

**Supplementary Information:**

The online version contains supplementary material available at 10.1186/s12936-025-05590-5.

## Background

Malaria continues to represent one of the leading causes of death in children globally, with an estimated 263 million cases of malaria and 597,000 deaths in 2023 [1]. Sub-Saharan Africa continues to carry a disproportionately high share of the global malaria burden, accounting for more than three-quarters of deaths in children under five.

In most malaria endemic countries, a range of interventions have been implemented, including: widespread deployment of insecticide-treated nets (ITNs); the use of indoor residual spraying (IRS) of insecticides in limited settings; chemoprevention for pregnant women and children; prompt diagnosis using quality-assured rapid diagnostic tests (RDTs) or high-quality microscopy; and treatment with highly effective artemisinin-based combination therapy (ACT), including improved access at the community level.

Despite extensive malaria control interventions, malaria remains a significant threat. The RTS,S malaria vaccine has been shown to reduce both uncomplicated and severe malaria in clinical trials. In 2015, the World Health Organization (WHO) recommended pilot implementation to explore the safety, feasibility, and impact of introducing RTS,S through routine vaccination programmes.

Ghana, Kenya, and Malawi were the first countries to introduce the malaria vaccine into their childhood immunization systems in 2019 as part of a pilot programme called the Malaria Vaccine Implementation Programme (MVIP) [2]. Early evaluation of new vaccine programmes is essential to allow for improvement and ensure high coverage and impact of a new vaccine. Post-introduction evaluations (PIE) were conducted in the three countries between May and October 2021, at least 2 years after the introduction of the vaccine. The World Health Organization (WHO) recommends the PIE [3] be conducted 6–12 months after any new vaccine introduction, allowing countries to evaluate activities within the immunization programme at all levels of the system using a standardized set of tools. Most countries have experience conducting PIEs following the introduction of new vaccines. The PIE aims to identify best practices and lessons learned to enable course correction and improvements to the programme during the critical early period of introduction. PIEs typically take around 10 days to implement and require a field team to travel to selected sites, conducting a series of standardized interviews with immunization experts and service providers at all levels of the health system, as well as observing vaccination sessions and vaccine storage sites. The PIEs took place later than is typically recommended in the pilot countries due to delays related to the COVID-19 pandemic. Beyond the PIE, the MVIP included a multi-faceted evaluation of the RTS,S vaccine pilot implementation led by in-country research partners. Results from these additional evaluation modalities are reported elsewhere [4].

The World Health Organization (WHO) recommends a 4-dose schedule of the malaria vaccine in children from 5 months of age to reduce the disease and burden of malaria [5]. To achieve prolonged protection, the fourth dose should be given 6–18 months after the third dose. To improve coverage, there can be flexibility in the timing of the fourth dose, including aligning it with vaccines given in the second year of life. Along with the introduction of vaccines, national malaria control programmes should ensure that existing WHO-recommended prevention tools continue to be deployed in accordance with national and sub-national policies. Multiple characteristics of the malaria vaccine programme were novel compared to other childhood vaccines (e.g., four doses in the schedule, new vaccination time points for children, including a dose administered in the second year of life), making the programmatic evaluation process particularly important. The PIE can be used to: (1) highlight deployment activities that went well and should be maintained; (2) identify gaps needing corrective action; (3) highlight lessons learned from vaccine deployment to strengthen the country’s overall national immunization system and services; (4) inform recommendations to improve roll-out of malaria vaccines; and (5) provide lessons learned for other countries for their malaria vaccine deployment and future deployments of other new vaccines.

The WHO provided scientific and technical leadership for the MVIP and coordinated the programme in close collaboration with PATH, GSK, UNICEF, the Ministries of Health of the three participating pilot countries, and country-based research partners. Through the MVIP, the RTS,S malaria vaccine was provided to children in the three countries in selected sub-national areas of moderate to high *Plasmodium falciparum* transmission. The implementation areas chosen had year-round moderate to high malaria transmission (with a parasite prevalence of greater than 20% in children under 5 years of age), as well as good immunization and malaria programme structure and functionality. The purpose of the MVIP was to assess the feasibility, safety, and impact of the vaccine in the context of routine use alongside other ongoing recommended malaria control measures.

In Malawi, Ghana, and Kenya, RTS,S was introduced in April, May, and September 2019, respectively. The malaria vaccine schedules selected in each country are shown in Table 1. Additional information about the subnational areas selected for vaccine introduction, planning, and implementation of vaccine roll-out, and methods for the MVIP evaluation are described in the accompanying implementation paper [6].

**Table 1:** Malaria vaccine implementation roll-out date and schedule in Ghana, Kenya, and Malawi

Country	Malaria vaccine implementation roll-out dates	Schedule
Ghana	May 2019	First three doses: 6, 7, 9 months–4th dose at 18 months*
Kenya	September 2019	First three doses: 6, 7, 9 months–4th dose at 24 months
Malawi	April 2019	First three doses: 5, 6, 7 months–4th dose at 22 months

*Ghana initially provided dose 4 at 24 months, but revised to 18 months in March 2023 in the context of vaccine expansion to MVIP comparator areas

This paper describes the methods and findings of the PIEs conducted in the three pilot countries. The paper also outlines how the results were used to improve malaria vaccination during the pilots and to enhance malaria vaccine introduction activities in the rollout to countries following the end of the MVIP.

## Methods

### Tools adaptation

The WHO generic PIE tools [3] were adapted to address the specific challenges and issues associated with the introduction of malaria vaccines. Each of the three countries further adapted the tools to their local context during the preparatory period for the activity. Based on the recommended methodology, PIEs were performed at all levels of the health system, including central/national, regional/county or district/sub-county, and health facility levels. Semi-structured interviews were conducted, featuring both quantitative and qualitative questions, during field visits at the national, sub-national, and health facility levels, as well as with specific target groups. These interviews were supplemented with systematic observations of vaccination sessions and vaccine storage sites. Six semi-structured questionnaires were used: (1) National level; (2) Regional/district level; (3) Health facility level; (4) Vaccine storage observation; (5) Vaccine session observation; (6) Caregiver interview at the health facility. Vaccine storage observations included verification of the appropriate and functioning cold chain equipment, as well as examination of vaccine vials, including vaccine vial monitors (VVMs). VVMs are small indicators that adhere to vaccine vials and change colour as the vaccine is exposed to cumulative heat, allowing health workers to know whether the vaccine has exceeded a pre-set limit beyond which it should not be used. Caregiver interviews consisted of yes/no and open-ended questions.

### Planning and site selection

PIE planning is conducted by a core lead implementation team, composed of members from the Ministry of Health and including experts in immunization and malaria programmes. A minimum and maximum number of site visits at each level of the health service allows a comprehensive overview of the system (Table 2). The maximum number of sites selected depends on the country’s size, the heterogeneity of its health and vaccination services, and the availability of human and financial resources to conduct the evaluation. Typically, one immunization staff member is interviewed at each facility visited.

**Table 2:** Recommended number of interviews by health administration level

Health administration level*	Minimum number of interviews
Central level	1
Regional/provincial level	3–6
District level	6–12
Health facility level	18–36

*Some countries may only have three administrative levels, and some may have more

The selection of which regions, districts, and health facilities or vaccination sites to evaluate was based on the country context. Some hard-to-reach sites were included to ensure the evaluation was more geographically representative and to account for equity issues. Sites selected represented a range of estimated vaccine coverage. Selection criteria identified a mix of sub-national areas based on immunization programme performance, geographic and ethnic diversity, serving underserved or at-risk populations, and size and type of facilities (urban, rural, fixed post, outreach). A convenience sample of caregivers was interviewed at the health facilities among those who presented for immunization services. Caregivers were asked to participate after being provided a verbal explanation of the interview’s purpose in their local language.

The PIEs were completed within approximately 10 days. An overview of a typical 10-day evaluation timeline is outlined in Table 3.

**Table 3:** PIE implementation timeline

Days 1–2	▪ Team meeting to review objectives and ensure consistent interpretation of PIE tools ▪ Adaptation of tools for the country situation ▪ Meet with the Ministry of Health (MoH), officials of the Essential Programme on Immunization (EPI), and key partners
Day 3	▪ Travel to the field–preferably at the weekend if travel times are long
Days 4–7	▪ Field visits to the region, districts, and health facilities
Days 8–9	▪ Return from the field ▪ Data compilation and analysis ▪ Writing a report and recommendations
Day 10	▪ Reporting to the MoH and the Inter-agency Coordinating Committee (ICC) ▪ Finalization of report and recommendations

The Ministry of Health leads the PIE in each country, with a team lead and members with strong knowledge of immunization and malaria vaccine programmes, their target populations, programme monitoring, and data analysis. A mix of external participants are invited from all levels of the WHO (global, regional, and country-level) and UNICEF, as well as other key in-country immunization partners, such as PATH or John Snow International (JSI), and non-governmental organizations active in the malaria vaccine programme.

### Field data collection

In each country, the evaluation team consisted of officers from the Ministry of Health’s EPI, the National Malaria Control Programme, and partners from WHO, PATH, and UNICEF. In Malawi, partners from JSI were also involved.

### Data analysis and reporting

In all three countries, the Open Data Kit (ODK) software platform was used to capture data electronically in the field. Upon completion of fieldwork, questionnaire data were summarized using descriptive statistics in MS Excel, EpiInfo, and STATA. The District Health Information Software was the source of administrative vaccination performance data used for selecting field sites and for reporting indicators. Key findings were shared with government stakeholders and key local partners for discussion and development of recommendations for programme improvement. A comprehensive report was developed to document the results and conclusions of each country.

## Results

### General impressions of malaria vaccine introduction

Feedback from national and subnational interviews in all three pilot countries suggested that vaccine introduction was smooth and was thought to improve immunization services (Table 4).

**Table 4:** MVIP pilot country post-introduction evaluation (PIE) characteristics

	Ghana	Kenya	Malawi
PIE start date	29 September–8 October 2021	8–16 August 2021	20 May to 2 June 2021
Time from vaccine introduction start to PIE start	30 months	24 months	26 months
Vaccine topics	Malaria	Malaria, HPV, Yellow Fever	Malaria
Sampling method	Purposive	Purposive	First stage random selection of regions (“zones”), second stage purposive
Additional site selection criteria	• Geographic representation • Urbanization (urban, peri-urban, rural) • Immunization coverage, malaria vaccine dose 1 (MV1)	• Geographic representation • Urbanization status (urban, peri-urban, rural) • Range of Immunization coverage, third dose pentavalent vaccine (Penta3), third dose malaria vaccine (MV3), and dropout rates	• Geographical representation • Range of immunization coverage
N regions	3	4 (counties)	3 (zones)
N districts	18	12 (sub-counties)	6
N health facilities	54	24	18
N caregiver interviews	94	25	32

When health workers (HWs) at the facility level were asked if the malaria vaccine introduction process was smooth in each of the three countries, 56, 79, and 67% responded positively in Ghana, Kenya, and Malawi, respectively (Table 4). In Kenya, 93% of HWs believed it improved the overall immunization service, compared to 56% in both Ghana and Malawi.

Ghanaian immunization staff reported subjectively that the introduction of RTS,S made an impressive impact on the under-five malaria case burden, improved immunization coverage, especially for second-year-of-life vaccines, reduced default rates, and resulted in a general increase in individuals seeking healthcare. The childhood immunization programme had been strengthened through improved vaccine supply, logistics, surveillance, advocacy, communication, and quality of service delivery.

Qualitative health worker responses aligned with quantitative results. A Ghanian interviewee (national level staff) stated:

*“We did not encounter any challenges in the roll out except that uptake in the initial phase was poor due to hesitancy. However, with continual engagement, this has improved”.*

Supporting the finding that vaccine introduction improved immunization service delivery, a regional-level officer said:

“*Some of our facilities did not have vaccine fridges and motor bikes but thanks to MVIP these logistics have been made available*.”

Another district level staff shared a positive effect of malaria vaccine introduction:

*“The number of malaria cases recorded monthly in this district has reduced by about one-fifth. Caregivers are seeing the impact and are more encouraged to vaccinate their children”.*

In Kenya, reasons for reporting an improvement in immunization services were numerous, including: more children coming to the health facility for malaria vaccine that are given other antigens; increased uptake of the second dose of measles rubella vaccine (MR2); increase in Vitamin A uptake at 6 months; reduction in hospital visits and blood transfusions due to malaria; reduction in hospital admissions of severe malaria cases; and health services having a variety of services to offer. The following quotes from interviewees in Kenya attest to these noted improvements:

A Kenyan health worker at the sub-county level said*:*

*“Since the malaria vaccine was introduced, our Vitamin A [coverage] at 6 months has improved; previously, the majority used to present at 9 months when coming for the MR Vaccine”.*

Another sub-county level nurse noted*:*

*“Since we have started to give malaria vaccine dose 4, our MR2 numbers have increased. Since we capture at 24 months those who didn’t turn up at 18 months at 24 months.”*

The medical superintendent at one of Kenya’s sub-county hospitals said*:*

*“Since we have introduced the malaria vaccine, we have seen a reduction in severe malaria cases requiring admissions, although we still get outpatient cases”.*

In Malawi, interviewees observed an improvement in the immunization service and proposed several reasons, noting that since the vaccine was introduced, the quality, consistency, and frequency of supervision had increased. It was also noted that the introduction of the malaria vaccine through the pilot programme brought more resources, improved AEFI surveillance, increased caregiver participation in immunization services, and facilitated the screening of children for other antigens during the malaria vaccine screening process.

### Pre-implementation planning

In all three countries, malaria vaccine introduction plans elaborated before introduction were cited as providing a foundation for pre-implementation planning. There was evidence of strong collaboration between the national immunization programme and other health programmes, including the malaria programme, at all administrative levels (national, regional, and district). Collaboration with key stakeholders and international partners was also highlighted as a key contributor to the successful introduction of malaria vaccines. In Malawi, the establishment of a national task force before the introduction, which met monthly, was identified as essential to guide preparations for and the implementation of the malaria vaccine introduction.

Challenges were reported, including a lack of district involvement in the early planning phase and delays in implementing social mobilization and demand generation activities. Activities such as the orientation of local leaders, caregivers, peer-to-peer engagement, and media engagements were all implemented after the vaccine was introduced and would have been more effective had they been conducted before its introduction.

### Coverage, recording, and reporting

**Fig. 1:**
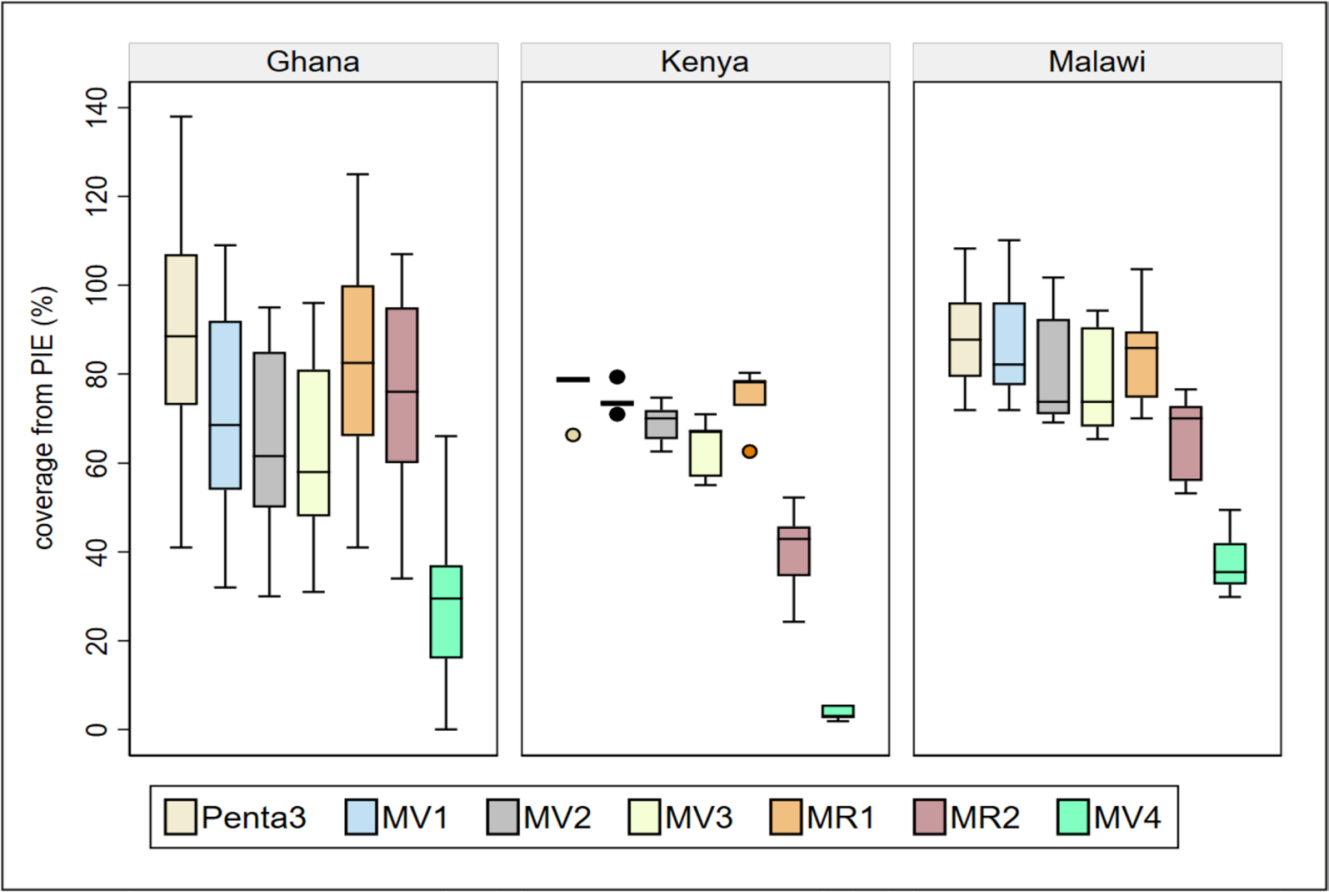
Malaria vaccine coverage* for the three pilot countries (Ghana, Kenya, Malawi) based on data collected in the post-introduction evaluation. *Coverage presented represents the median and interquartile range (IQR) based on monthly administrative coverage reported for 12 months in selected health facilities for 18 districts in Ghana and five sub-counties in Kenya between January and December 2020. For Malawi, coverage was based on reported administrative data, selecting health facilities (HFs) in 12 districts for a 6-month period between October 2020 and March 2021. Abbreviations: Penta3, third dose pentavalent vaccine; MV1-4, first through fourth dose malaria vaccine; MR1-2, first and second dose measles and rubella containing vaccine

**Fig. 2:**
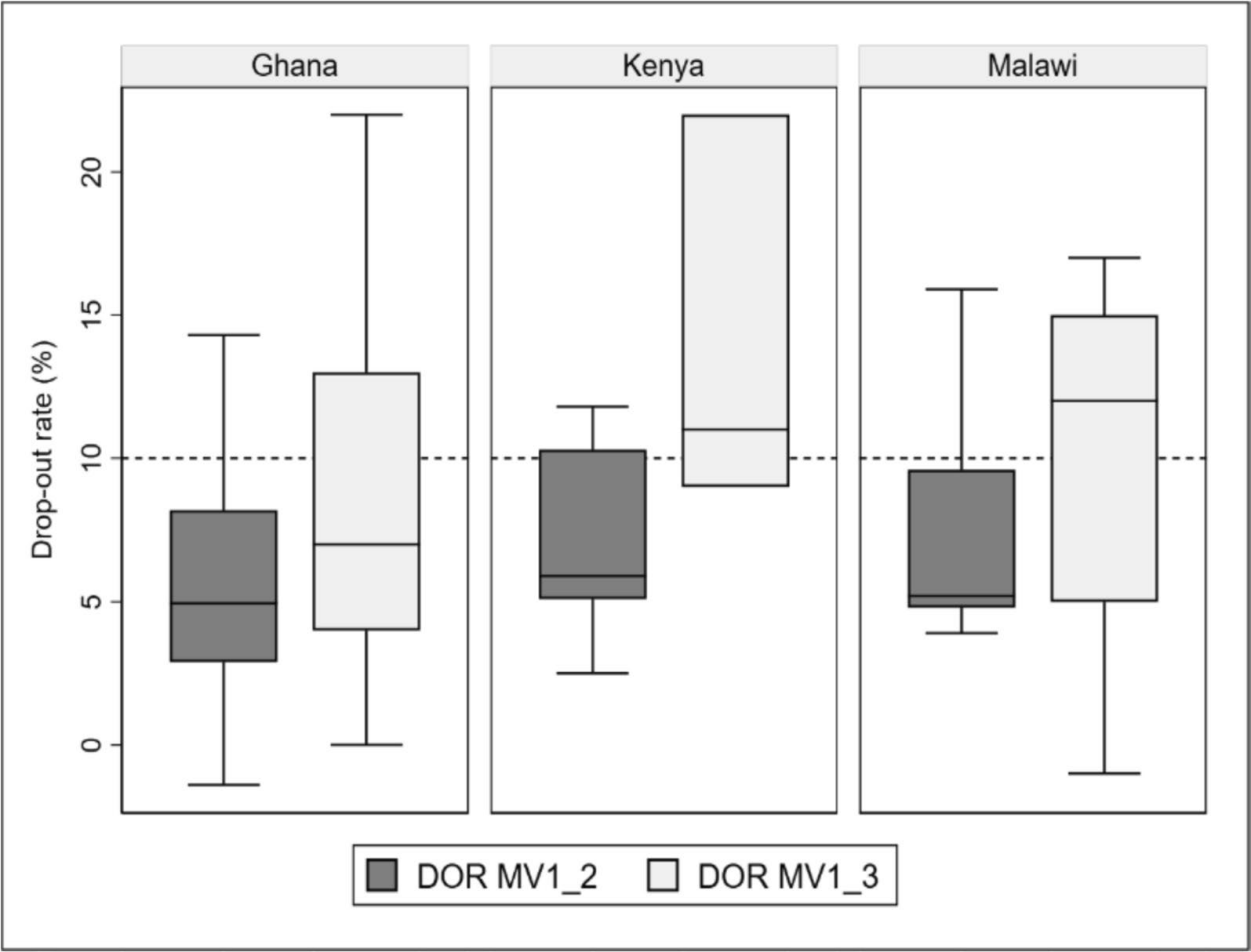
Drop-out rates between the first dose malaria vaccine and the second (DOR MVI_2) or third dose (DOR MV1_3) malaria vaccine for Ghana, Kenya, and Malawi

Coverage and drop-out rates for all four doses were reported as part of the PIE for the period from January to December 2020 for both Ghana and Kenya, noting that Ghana initiated vaccine implementation 5 months earlier than Kenya. Malawi opted to report 6 months of coverage data from October 2020 to March 2021 for all doses (Figs. 1 and 2, Table 5). The drop-out rate is the difference in the percentage of children who start a vaccine schedule and those who complete it.

**Table 5:** Key malaria post-introduction evaluation (PIE) findings across the three pilot countries: perceptions of health workers and caregivers

Respondent	Key findings	Ghana	Kenya	Malawi
Immunization staff perceptions	Introduction improved the EPI programme	62.8% (49/78 Regional, district, and facility staff)	100% (12/12 County and sub-county staff)	83% (5/6 District staff)
	Introduction improved EPI	56% (30/54)	93% (13/14)	56% (10/18)
	Introduction was smooth	56% (30/54)	79% (11/14)	83% (15/18)
	Accepted as an additional malaria control tool	88% (47/54)	100% (12/12)	100% (18/18)
Caregiver knowledge and perceptions	Heard about vaccine	93% (87/94)	96% (24/25)	97% (31/32)
	Accepted the vaccine	96% (90/94)	100% (25/25)	83% (15/18)
	Vaccine knowledge (4 doses for maximum protection)	34% (32/94)	60% (15/25)	46.9% (15/32)
	Knew when to return to receive the next dose	66% (62/94)	96% (24/25)	83% (15/18)
	Aware of potential common side effects following vaccination	83% (78/94)	84% (21/25)	83% (15/18)
	Satisfied with the services received	98% (92/94)	88% (22/25)	100% (32/32)

In all three countries, MV1 and MV3 coverage for the reported period was lower than coverage for the first and third doses of the pentavalent vaccine (Penta1 and Penta3, respectively) at all administrative levels. Coverage decreased consistently from the first dose to the fourth dose; however, data for the fourth dose were very limited for all countries during the reporting period for the PIE. In all countries, the coverage of the third dose of the malaria vaccine remained low compared to that of the measles first dose, which is given at the same time.

The three countries reported a drop-out between MV1 and MV3 and between MV2 and MV3. Although drop-out data were also reviewed between MV3 and MV4, countries had not had enough time to provide MV4 for the results to be informative. Median first-to-third (MV1_3) dose drop-out rates were 7% (4–13%) in Ghana, 11% (9–22%) in Kenya, and 12% (5–15%) in Malawi (Fig. 2). The first-to-third drop-out rates varied by subnational level. In Kenya, the rates range from 9 to 22%; in Malawi, from − 1%% to 17%; and in Ghana, from 0 to 22%. The dropout rates were higher between the first and third dose compared to those between the first and second (Fig. 2).

While the majority of staff in all countries knew the correct formula for calculating vaccination coverage and knew the population of their catchment area, HWs at the regional or health facility level believed that the target population figures were inaccurate (Ghana, 37%, Kenya 43% and Malawi 11%), raising questions over the accuracy of the data reported for vaccine coverage.

### Recording and reporting

Monthly data reporting was generally good, with 83% of facilities in Ghana, 98.6% of facilities in Kenya and 94% in Malawi reporting data monthly, and 88.9% of districts in Ghana, 100% of districts in Kenya and 94% of health facilities in Malawi achieving both data completeness (i.e. all facilities report) and timeliness (i.e. a facility provides a report within a month) of at least 90% in 2020.

Recording and documentation of malaria vaccination was observed to be accurately done in most facilities. Where available, coverage monitoring charts that include the malaria vaccine were up to date, displayed in the health facility, and staff knew how to interpret the data. However, in some districts and health facilities in Ghana, coverage monitoring charts were either unavailable or not displayed. In instances where they were available, the malaria vaccine was often not included.

In Kenya, in general, updated recording and reporting tools were available at the health facility level. There were, however, reports of gaps in the availability of permanent registers (79%), eligibility criteria tools (71%), and mother and child health books (71%). Some facilities were using exercise books to record vaccinations due to stockouts of vaccine stock record books. Monitoring charts for the malaria vaccine were not provided; however, in 36% of health facilities, staff had improvised coverage monitoring charts to track the performance of the malaria vaccine. Similarly, in Malawi, the vaccine stock record book had not been updated to include the malaria vaccine, and late distribution of reporting tools was observed in most facilities. Only 44% of the health facilities had malaria vaccine monitoring charts, and the remainder were unable to monitor malaria vaccine uptake easily. Similarly, in Malawi, there was a delay in printing and distributing the revised data recording and reporting materials, including the under-2 registers, monthly reporting forms, and malaria vaccine monitoring charts. The majority of health facilities reported receiving these tools long after the vaccine had been introduced.

### Cold chain management

In Ghana, 83% of health facilities had functional refrigerators, and all regional and district cold stores had fridge tags for continuous temperature monitoring. In most facilities with functional refrigerators, vaccines were appropriately arranged, temperatures were recorded twice daily, including weekends, and vaccines were stored at temperatures between + 2 °C and 8 °C. Staff reported that vaccines were transported in cold vans equipped with built-in temperature monitoring devices.

In Kenya, all health facilities visited had functional refrigerators and vaccine carriers, and 86% had functional fridge tags. However, one weakness noted for HWs was that none of the sub-counties used fridge tags during the transportation of vaccines.

In Malawi, 94.4% of health facilities visited had functional refrigerators, fridge tags, and vaccine carriers. All health facilities visited had temperature monitoring charts, and in the majority (88.9%) of health facilities observed, temperatures were recorded twice a day, including weekends and holidays. Temperatures ranged from + 2 °C to 8 °C in 96.3% of the facilities visited.

Both Ghana and Kenya needed to enhance their cold chain capacity before introducing the malaria vaccine to accommodate it. Despite this investment, some problems persisted. For example, the procurement of non-standard fridges and the lack of functional refrigerators in 16.7% (9/54) of facilities in Ghana, as well as reports of inadequate cold chain capacity in 2 out of the 4 counties sampled in Kenya at the administrative level. Ghana needed to increase vaccine shipments from twice a year to once a quarter, which had additional financial implications for the country.

### Vaccine management, transport, and logistics

In Ghana, the majority (96.2%) of health facilities had no expired vaccines or vaccines at VVM stages 3 or 4 in the 6 months preceding the PIE. A large proportion of the staff interviewed (90.7%) reported verifying both the VVM and the expiry date before using the vaccines. One region and two districts in Ghana reported stockouts of RTS,S and supplies in the 6 months preceding the PIE, and approximately 24% (13/54) of facilities reported stockouts of RTS,S and other EPI vaccines.

In Kenya, there were no stockouts of the malaria vaccine in 88% (7/8) of sub-counties, and 93% of the health facilities visited had not experienced any vaccine stockouts in the 6 months preceding the PIE. Furthermore, HWs knew which vaccines to use first based on expiration dates and VVM status. Although no health facilities had expired vaccines at the time of the evaluation, there were reports of expired malaria vaccines in the past due to the short shelf life of the vaccine.

Gaps were observed in Kenya’s documentation in the vaccine ledgers, where facilities (number not quantified) often lacked entries for diluent information, as well as some delays in recording vaccine information. In Malawi, most health facilities did not record the malaria vaccine in their vaccine stock records, as there was no dedicated space for recording it.

In Malawi, no serious stockouts or delays in the provision of malaria vaccines were reported in the 6 months preceding the evaluation. Malawi experienced issues with the transportation of vaccines from the national store to the regional level, resulting in negative consequences for immunization services in some health facilities. Additionally, some HWs interviewed reported that outreach clinics in certain health facilities were not well-supported with fuel and maintenance of motorcycles by their district health management teams. Additionally, 33% of the districts visited had limited storage space for injection materials.

### Training and knowledge of healthcare workers

In all three countries, many HWs participated in some form of training or orientation before the introduction of the malaria vaccine. Malawi reported that 100% of health facility staff were trained before the introduction of the malaria vaccine. However, in Ghana, only 65% of staff had been trained before the introduction of the vaccine. In Kenya, only 43% of those interviewed at the facility level had been trained, as only two staff members were trained in each facility, and staff turnover was high. HWs were confused about the eligibility criteria, primarily due to a policy change that extended the age range eligible for vaccination to include older children. This change occurred late in training and may not have been well-articulated. Furthermore, in Kenya, it was noted that the sub-county medical officers were not invited to the training despite being the team leads at the sub-county level.

Nevertheless, in all three countries, HWs showed good knowledge of the malaria vaccine schedule and eligibility criteria (except for the confusion mentioned in Kenya), as well as guidance on the steps to follow for a child who presents late for vaccination. Additionally, HWs were able to describe the correct recording procedures. During observation of vaccination sessions, HWs in all three countries proactively reminded caregivers of the return date, of the importance of continuing to use other malaria prevention interventions, and of the importance of completing the immunization schedule. In all countries, staff who attended training had the opportunity to practice administering the RTS,S vaccine correctly with dummy vials made available.

While job aids, training materials, quizzes, WhatsApp information, and other Information, Education, and Communication (IEC) tools were in use, they were not consistently available across the three countries, nor were they consistently available within the same country.

### Monitoring and supervision

In the three countries, most health facilities (Median 87%: Ghana 87% (47/54), Kenya 57% (8/14), Malawi 100% (18/18)) had received at least one supervisory visit in the 6 months preceding the PIE and received feedback from the supervisors. In many cases, feedback was provided verbally rather than in writing. Feedback was written in 17.0% (8/47) of facilities in Ghana, 88% (7/8) in Kenya, and 58% (11/19) in Malawi.

### Advocacy, communication, and acceptance

Before the introduction of malaria vaccines, a communication strategy was developed, and social mobilization activities were conducted at the sub-national level in all three countries. Communication materials, including flipcharts, posters, and booklets on the malaria vaccine, were developed by adapting WHO information, education, and communication materials and disseminated to the service delivery level.

While social mobilization activities were conducted before implementation, both Ghana and Malawi noted that these were insufficient to dispel various concerns, and up to 64% of health workers (in Kenya) had experienced vaccine refusals. In Malawi, only 11% of the health facilities evaluated had encountered refusal or resistance to the RTS,S malaria vaccine. Reasons for hesitancy or refusal included rumours that the vaccine was being trialed.

In Ghana, the malaria vaccine was reported to be acceptable among most healthcare workers, professionals, and community members; however, approximately 50% of healthcare workers interviewed had experienced incidents of RTS,S refusals. Misinformation was cited as a major concern for low vaccine uptake, due to the proliferation of smartphones and social media that facilitated the spread of misinformation ahead of the vaccine’s launch. In Kenya, 64% of health facility staff reported refusal of the malaria vaccine, and this was partially attributed to the delayed sensitization of community health volunteers.

The majority (> 80%) of caregivers interviewed in all three countries reported that they had heard about the malaria vaccine from their healthcare provider, and most (66–96%) knew when to bring their child in for the next dose of the vaccine (Table 4). When asked about possible side effects of the malaria vaccine, they were able to name at least one potential side effect correctly. In many cases (66.7%) of mothers interviewed in Malawi, knowledge of Adverse Event Following Immunization (AEFI) prompted them to return to the hospital to report and receive the correct treatment.

**Fig. 3:**
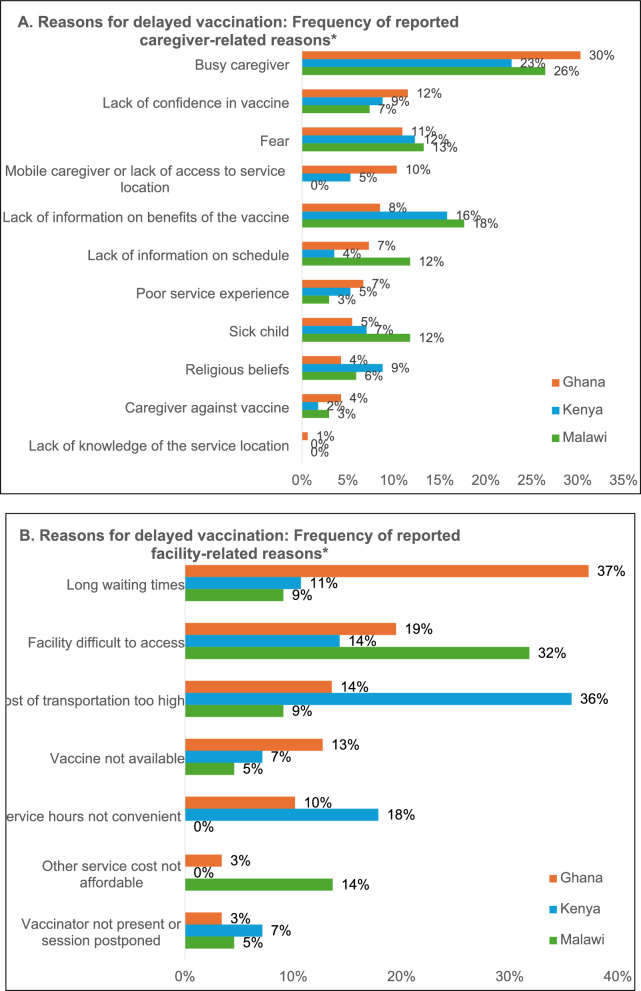
Frequency of (**A**) caregiver and (**B**) facility-related reasons for delayed vaccination as reported by caregivers. *Caregivers were allowed to respond openly and could provide multiple responses, which were categorized. Responses are not mutually exclusive. Total caregiver-related responses: Ghana, N = 165, Kenya, N = 57, Malawi, N = 68. Total facility-related responses: Ghana (N = 118), Kenya (N = 28), Malawi (N = 22)

The most common reasons caregivers provided for the delay in malaria vaccine doses in their community were that caregivers were too busy, followed by a lack of information on the benefits or fear of the vaccine (Fig. 3a). The most commonly reported facility-related factors varied by country (Fig. 3b). In Ghana, long waiting times were cited most often as problematic. The cost of transportation was most commonly reported in Kenya, and difficulties accessing facilities were most commonly reported in Malawi.

### Injection safety and waste management

In all three countries, all health facilities visited were using safety boxes for the disposal of auto-disable syringes. In all three countries, around 80% of the used safety boxes were disposed of appropriately. Some health facilities in both Ghana and Malawi were burying or using open-pit burning of the safety boxes due to the unavailability of incinerators or closed pits for burning the waste material.

### Vaccine wastage

In all three countries, over 80% of health workers (HWs) were aware of the correct formula for calculating vaccine wastage rates and reported this information every month. In Ghana, 88% of districts had RTS,S wastage rates within an acceptable range, up to 25%. In Kenya, the average wastage rate was between 8.9% and 10%. However, in Malawi, 56% of health facilities had wastage rates above the recommended figure for all antigens, most notably for Bacillus Calmette Guérin (BCG) and measles-rubella (MR) vaccines.

### Safety monitoring

Most of the staff interviewed had received training on AEFI in the last 6 months and knew the correct definition for AEFI. In Kenya and Malawi, national AEFI monitoring and reporting guidelines were available in more than 80% of the facilities visited, whereas in Ghana, this was the case in only 55.6% of facilities.

In Kenya, all counties were prepared for risk communication in the event of an AEFI related to the malaria vaccine. In Malawi, health workers (HWs) reported that the introduction of the malaria vaccine had increased their knowledge of adverse events following immunization (AEFI) and the reporting of vaccine safety issues, which had improved since the introduction of the malaria vaccine due to heightened awareness of the importance of reporting.

### Financing and sustainability

In all three countries, there were financial implications linked to the introduction of RTS,S. Although funding was provided by both the MoH and MVIP partners in all three countries, there were discrepant responses from interviewees at both national and subnational levels when asked about their understanding of the source of funding. Interviewees at the subnational level in all three countries reported that the MVIP partners primarily financed the introduction of the program, without additional resources from the MoH. This perception was related to the fact that, at the health facility level, the addition of a malaria vaccine had minimal implications for operational costs, as it was integrated with existing immunization service delivery platforms supported by the MoH (i.e., the malaria vaccine is delivered with other antigens and not through separate sessions).

In Ghana, despite the availability of partner funding, 9% of facilities reported having to cancel some critical routine immunization activities due to a lack of funds. A field technician said:

*“Sometimes we are unable to get money to purchase fuel and have to abandon outreach services. This results in default, especially among children living in the hard-to-reach communities”.*

Similarly, at the national level in Malawi, it was noted that there were insufficient funds to implement all planned activities thoroughly and promptly for introduction. Facility-level responses were not available from the Malawi interviews on this topic. In Kenya, the planning and introduction of the malaria vaccine were reported as having been well-resourced, in contrast to other new vaccine introductions. However, this was understood as a pilot process, and no particular concerns were raised regarding sustainability.

## Discussion

Conducting PIEs during the malaria vaccine pilot implementation period has been important not only for improving malaria vaccination programmes in Ghana, Malawi, and Kenya during the pilot period but also for enhancing delivery as the vaccine was expanded to comparator areas and beyond. The documented lessons learned from the MVIP PIEs and other forms of evaluation from the pilot continue to inform malaria vaccine introductions in multiple endemic countries, starting in 2024. As of August 2025, 22 countries have introduced a malaria vaccine, and at least three more plan to introduce it in 2025.

The lessons learned from the PIEs have been many, given the depth of the evaluation at all levels of the immunization system and across programme areas. Common best practices identified across the three countries include:

- **Early involvement of subnational levels** from the early planning phase of the new vaccine introduction should be encouraged to develop detailed subnational plans and budgets, which will improve the rollout of the vaccine at the service delivery level
- **Ensure update of all recording and reporting tools** to include the new vaccine, and then distribute to all administrative levels before the new vaccine is introduced
- **Pre-assessment of cold chain capacity** and scale up as needed before introducing the new vaccine is key to improving vaccine supply and management
- **Investment in HW training**s in terms of sufficient time, resources, and ensuring that all key health workers at all levels are trained to optimize impact
- Plan for **refresher training** with the expectation of staff turnover and the need to readdress priority topics
- Early training and** involvement of community health volunteers**
- Ensure **robust defaulter tracing mechanisms** are in place as an essential tool in reducing vaccine dropout rates
- Ensure a **“dialogue” with the community** through continuity of advocacy, communication, and social mobilization activities after the initial introduction phase, to reduce dropout rates and ensure children complete the four-dose malaria vaccine immunization schedule.
- Conduct **regular onsite supervisory visits** with written feedback before, during, and after introducing the new vaccine to improve and consolidate staff capacity in deploying and administering it.
- In light of the proliferation of misinformation and anti-vaccination sentiments, **rumors and misinformation must be actively addressed** using social media, key influencers, or other methods to spread the correct messages before introduction

While some of the challenges identified through the malaria vaccine PIE findings are similar to those from PIEs related to other new childhood vaccine introductions [7, 8], the malaria vaccine’s four-dose schedule, extending into the second or third year of life, has presented new challenges. The importance of both HWs and caregivers knowing when a child should return for a subsequent dose is paramount to the success of the malaria vaccine programme. Although many health workers were well-trained on the vaccination schedule, the training did not reach all HWs and when caregivers were asked if they knew when to return for the next dose, not all were well-informed. In areas where training of HWs is lacking, supportive supervision is a critical activity that can fill gaps where a staff member missed the initial training or where new staff were hired after the introduction. While interviews revealed that most facilities received at least one supervisory visit in the 6 months preceding the PIE, increasing the frequency of supportive supervision and ensuring clear, written feedback to staff has great potential to improve programme performance. In all three countries, efforts were made to increase supportive supervision or implement refresher training following the PIE. In Kenya, feedback was provided at all levels, and actions were integrated during continuing medical education (CME), supportive supervision, and mentorship. In Malawi, stakeholders implemented quarterly supervision following the PIE to strengthen supervision and mentorship. Periodic review meetings were also held, allowing district-level health personnel to learn from one another by sharing best practices, challenges, and solutions.

Coverage and drop-out rates reported during the PIEs described span a period of approximately 1–2 years after introduction and include data from all four doses, as well as in relation to other routine childhood vaccines. Although administrative coverage and dropout data were continuously monitored and reviewed since the introduction, systematic review and documentation of these national and subnational data during the PIE permitted reflection on trends in each of the three countries and continued response to low coverage and subnational variability. With the introduction of new vaccines, coverage is expected to increase over time [9], especially when new vaccination time points are introduced, as seen with malaria vaccines. Given that drop-out rates of less than 10% are the typical benchmark for immunization programmes, all three countries attained acceptable levels of drop-out within the first 1–1.5 years of introduction, as reported in the PIE. Although some MV4 drop-out data were reported during the PIEs, the early period of implementation described by the PIE was not long enough to allow for meaningful commentary on rates of MV4 drop-out. The administrative coverage of the fourth dose has been closely monitored over time in each of the pilot countries since the PIE was conducted.

Given the observed coverage and dropout rates in each of the three countries, activities were undertaken to reach more children to start vaccination and track defaulters. The periodic intensification of routine immunization (PIRI) is one strategy to increase communication in communities and increase outreach vaccination at both fixed and mobile sites. PIRs typically promote catch-up vaccination across all childhood vaccines, as well as the promotion of other malaria control measures.

In Ghana, PIRIs had been implemented before the PIE as part of measures to address low vaccine uptake, based on earlier analysis of routine data. The PIE provided further empirical evidence to justify the PIRI and other initiatives aimed at increasing demand and improving vaccine uptake. PIRs in Ghana were subsequently conducted when specific funding became available or when opportunities arose to leverage funding available for other outreach activities (e.g., seasonal malaria chemoprevention) targeting the same population. PIRIs included: (1) caregiver sensitization at service delivery points, community information centres, social events such as religious gatherings, funerals, and community durbars; and (2) defaulter tracing to identify and vaccinate missed children, and active search and vaccination of eligible children (children who are eligible but have not yet received the first dose). Malawi also conducted PIRIs both before and after the PIE to improve vaccine uptake through mapping and outreach to unvaccinated and under-vaccinated children. Kenya undertook an integrated outreach and defaulter tracking programme in poorly performing areas in 2022 and 2023, based on the PIE findings and recommendations.

Among the three pilot countries, Kenya took the opportunity to conduct an integrated PIE for evaluation of malaria, Human Papillomavirus (HPV), and yellow fever vaccines. Integrated PIEs or full EPI reviews can be very useful for capitalizing on the resources invested in evaluation to identify successes and challenges of the immunization programme across multiple vaccines, especially those given at the same time points in the EPI schedule.

By the end of 2024, 5 years after the initial introduction of the malaria vaccine, more than 9 million children will have received vaccinations, with over 3 million malaria vaccine doses administered. Coverage in the three countries has steadily increased over time, and drop-out rates have continued to decrease. Key lessons learned from the PIEs and other forms of programme evaluation have been applied as the three pilot countries expanded malaria vaccine implementation to additional malaria-endemic areas within each country, based on the expanded WHO recommendation for use. Ghana, in particular, made changes to its national policy based on lessons learned: given challenges with fourth dose coverage, the decision was made to shift the scheduled timing of the fourth dose from 24 to 18 months of age. While concerns about low fourth-dose coverage had been observed before the PIE, the PIE process provided documentation of coverage trends and opportunities to discuss potential changes in strategies. Especially in non-pilot vaccine introduction settings, the PIE documentation and follow-up process can be crucial for a country’s advocacy for needed policy changes and to garner the resources necessary for additional programme strengthening. Since Ghana’s schedule change, coverage of the fourth dose has risen markedly over time. Malawi and Kenya have maintained the timing of their fourth dose at 22 and 24 months, respectively, and coverage has increased steadily over time, albeit at a slower pace than seen in Ghana. Additional African countries that have started introducing the malaria vaccine in 2024 have used the lessons learned from the country to inform their introduction plans. These lessons are valuable for the effective implementation of malaria vaccines, regardless of the specific malaria vaccine product. The WHO currently recommends two malaria vaccines, RTS,S and R21/Matrix-M (R21), for the prevention of *P. falciparum* in children living in malaria-endemic areas. Both vaccines have the same dose schedule, as well as other programmatic considerations regarding introduction into national immunization programmes.

The COVID-19 pandemic introduced challenges to the implementation of the PIEs in all three countries. All three countries had introduced the malaria vaccine between April and September 2019. Under normal circumstances, the PIEs should have been carried out six to 12 months after the vaccine introduction, i.e., during 2020. However, due to the COVID-19 pandemic at the time, it was not possible to organize such a wide-scale evaluation until 2021, as restrictions on internal travel in some countries, limitations on international partners’ travel, and difficulties in organizing meetings and workshops in various countries all contributed to the challenge. Additionally, considering the recommended 4-dose schedule for the malaria vaccine, there was a need to wait to conduct the PIE until after the 4th dose had been administered.

PIEs are designed to be relatively simple for any country to conduct within a short period without requiring statistical support; however, they are limited by their design, which uses purposive rather than randomized data collection. The fact that caregiver interviews are typically administered only at the health facility location (for logistical ease) would not provide a representative view of all caregivers’ perceptions, as those presenting for immunization are likely to be different from those who do not. Therefore, PIE results cannot be generalized, and data points cannot be statistically compared across countries. Unlike most new vaccine introductions, the MVIP included robust evaluation beyond the PIE to assess feasibility, effectiveness, and safety [10]. Population representative coverage surveys were conducted in each country at baseline and at two time points post-introduction. These allowed for a quantitative assessment of the impact and trends in malaria vaccine coverage, as well as coverage of other childhood vaccines and malaria control methods. A health utilization survey was also conducted to provide a comprehensive qualitative assessment, understanding the perceptions of community members and health workers regarding the introduction of malaria vaccines [11]. The RTS,S vaccine manufacturer, GSK, is conducting phase 4 studies to assess the vaccine’s impact and safety. The multiple evaluation modalities included in the MVIP provide a comprehensive view of successes and challenges associated with the introduction of malaria vaccines. In countries conducting a PIE under normal circumstances, additional forms of more representative evaluation may be considered in response to or as a supplement to the important findings of the PIE. For example, a community-level behaviour and social drivers survey might be warranted to follow up on observed lack of community awareness or misinformation related to malaria vaccine implementation.

## Conclusions

A malaria vaccine is an important intervention as part of a comprehensive malaria control programme in moderate and high transmission settings. Conducting a PIE in the early period after introduction is helpful to identify challenges, best practices, and lessons learned to improve performance. Coverage at new vaccination time points may take months to years to establish and stabilize as communities become aware of, understand, and learn when, why, and how to access the malaria vaccine. The vaccine can be introduced successfully as part of the childhood immunization programme with strong intersectoral collaboration and planning, involving both immunization and malaria stakeholders, as well as robust training and social mobilization efforts pre- and post-introduction. The MVIP experience provided a strong foundation to guide additional countries in introducing the malaria vaccine. Most new vaccine introductions do not benefit from a rigorous evaluation, like the MVIP. Nevertheless, the PIE is a well-known methodology that can be conducted relatively easily by countries to generate valuable insights into programmatic functioning and provide lessons to enhance ongoing implementation.

## Supplementary Information

Additional file 1.

## Data Availability

Data will be made available on request.

## Abbreviations

AEFI: Adverse Events Following Immunization
ACTs: Artemisinin-combination therapies
EPI: Essential Programme on Immunization
HW: Health worker
ITN: Insecticide-Treated Nets
ICC: Inter-agency Coordinating Committee
MV: Malaria Vaccine
MVIP: Malaria Vaccine Implementation Programme
MR: Measles Rubella vaccine
MenA: Meningitis A vaccine
MoH: Ministry of Health
NMCP: National Malaria Control Programme
ODK: Open Data Kit
Penta: Pentavalent vaccine
PIRI: Periodic Intensification of Routine Immunization
PIE: Post-Introduction Evaluation
RDT: Rapid Diagnostic Test
VVM: Vaccine Vial Monitor
WHO: World Health Organization
