# Supplementary material for: POST introduction evaluation (PIE) of the malaria vaccine introduced in three pilot countries (Ghana, Kenya, and Malawi) in 2021
Source: Malar J. 2025 Oct 14;24:333. doi: 10.1186/s12936-025-05590-5 (PMC12522374; doi:10.1186/s12936-025-05590-5)
Supplement: Supplementary file 1 — Additional file 1. [file 12936_2025_5590_MOESM1_ESM.docx]

**SUPPLEMENTAL DATA:**

**Table S1:**

|  | **Immunization Coverage* (Median (IQR)** | | | | | | |
| --- | --- | --- | --- | --- | --- | --- | --- |
|  | Penta3 | MV1 | MV2 | MV3 | MR1 | MR2 | MV4 |
| Ghana | 89% (73,11) | 69% (54,92) | 62% (50,85) | 58% (48,81) | 83% (66,10) | 76% (60,95) | 30% (16,37) |
| Kenya | 84% (84,85) | 79% (78,79) | 75% (70,77) | 72% (61,72) | 84% (78,84) | 46% (37,49) | 3% (3,6) |
| Malawi | 94% (85,10) | 88% (83,10) | 79% (73,97) | 79% (73,97) | 92% (80,96) | 75% (60,78) | 38% (35,45) |

*Coverage presented represents the median and interquartile range (IQR) based on monthly administrative coverage reported for 12 months in selected health facilities for 18 districts in Ghana and 5 sub-counties in Kenya between January and December 2020. For Malawi, coverage was based on reported administrative data, with selected health facilities (HFs) in 12 districts monitored for six months between October 2020 and March 2021.

Abbreviations: Penta3, third dose pentavalent vaccine; MV1-4, first through fourth dose malaria vaccine; MR1-2, first and second dose measles and rubella containing vaccine.
